# Novel factors contributing to fungal pathogenicity at early stages of *Setosphaeria turcica* infection

**DOI:** 10.1111/mpp.13140

**Published:** 2021-10-10

**Authors:** Yanan Meng, Fanli Zeng, Jingjing Hu, Pan Li, Shenglin Xiao, Lihong Zhou, Jiangang Gong, Yuwei Liu, Zhimin Hao, Zhiyan Cao, Jingao Dong

**Affiliations:** ^1^ State Key Laboratory of North China Crop Improvement and Regulation Baoding China; ^2^ College of Life Sciences Hebei Agricultural University Baoding China; ^3^ Key Laboratory of Hebei Province for Plant Physiology and Molecular Pathology Hebei China; ^4^ College of Plant Protection Hebei Agricultural University Baoding China

**Keywords:** appressorium, effector, pathogenicity, *Setosphaeria turcica*

## Abstract

The fungal pathogen *Setosphaeria turcica* causes leaf blight on maize, which leads to considerable crop losses. However, how *S. turcica* establishes sustained systemic infection is largely unknown. Here, we report several novel factors contributing to *S. turcica* pathogenicity, identified using a genomic and transcriptional screen at different stages of *S. turcica* appressorium development. We identified two cytoskeleton regulators, *SLM1* and *SLM2*, that are crucial for hypha and appressorium development. The *SLM1* and *SLM2* transcripts accumulated during germling stage but their levels were notably reduced at the appressorium stage. Deletion of *SLM2* dramatically affected cell morphology, penetration ability, and pathogenicity. We also identified three different types of *S. turcica* glycosyl hydrolases that are critical for plant cell wall degradation. Their transcripts accumulated during the appressorium infection stage induced by cellophane and maize leaf. Most importantly, we characterized a novel and specific *S. turcica* effector, appressorium‐coupled effector 1 (StACE1), whose expression is coupled to appressorium formation in *S. turcica*. This protein is required for maize infection and induces cell death on expression in *Nicotiana benthamiana*. These observations suggest that the phytopathogen *S. turcica* is primed in advance with multiple strategies for maize infection, which are coupled to appressorium formation at the early infection stages.

## Graphical abstract

The phytopathogen *Setosphaeria turcica* is primed in advance with multiple strategies for maize infection, which are coupled to appressorium formation at the early infection stages.

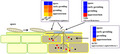

## 1 INTRODUCTION

*Setosphaeria turcica* is the causal agent of northern corn leaf blight, which is one of the most important maize foliar diseases (Cao et al., 2020; Perkins & Pedercens, 1987; Van Inghelandt et al., 2012). Early infection starts when a conidium of *S. turcica* lands on the maize leaf surface and germinates to form a highly polarized germ tube. The germ tube elongates and becomes flattened against the plant surface. Growth at the germ tube tip then ceases, and the tip swells isotropically, resulting in the formation of a dome‐shaped appressorium (Hilu & Hooker, 1964, 1965; Knox‐Davies, 1974). The appressorium generates high turgor, which is used by the fungus to create mechanical force for penetration of the plant cuticle and cell wall by physical force, and enables entry to the underlying epidermal cells (Gu et al., 2014; Knox‐Davies, 1974; Wang et al., 2005).

To initiate appressorium development, the fungus responds to a set of inductive cues, including surface hardness, hydrophobicity, cuticular wax, and the absence of exogenous nutrients (Ebbole, 2007; Ryder & Talbot, 2015). We have previously demonstrated that cAMP and protein kinase A signalling pathway, a mitogen‐activated protein kinase pathway, and an ATR (Ataxia Telangiectasia and Rad3 related)‐mediated S‐phase checkpoint are necessary for efficient appressorium morphogenesis and the subsequent invasive growth of *S. turcica* in response to environmental signals or stresses (Gong et al., 2017; Shen et al., 2013; Zeng et al., 2020). However, essential *S. turcica* factors that couple appressorium formation to the ensuing pathogenicity are still largely unknown.

During the early infection stages, fungi frequently switch between isotropic and polarized growth. This is intimately linked to the cell cycle regulation in yeast, and filamentous and dimorphic fungal species (Momany, 2002; Steinberg & Perez‐Martin, 2008). The essential genes controlling isotropic and polarized growth in *S*. *turcica* have not yet been identified, although we have previously shown that the upstream‐acting genes *STE12* and *Ras2* are related to hyphal growth and development (Gu et al., 2014; Knox‐Davies, 1974; Wang et al., 2005).

Polarized growth is characterized by a localized cell wall expansion at the growth tip, while isotropic growth pattern entails uniform cell wall expansion over the cell surface (Pruyne & Bretscher, 1928). Studies of pathogenic fungi have provided general insights into the regulation of growth of a germinating conidium. Polarized growth produces elongated cells, while isotropic growth produces round cells (Takeshita, 2016). Changes in the balance between polarized and isotropic growth are modulated by cytoskeleton modification, which leads to the variety of cell shapes observed during appressorium development and infection (Harris, 2011; Takeshita, 2016). A strong ability to activate polar growth for germination and to form a filament determines the success of the early infective process. Cytoskeleton regulators are hence deemed to be the central factors controlling the morphology of conidial germling and appressorium formation (Flor‐Parra et al., 2007; Momany & Talbot, 2017; Ryder & Talbot, 2015). As in other fungi, morphogenetic transitions mediated by cytoskeleton regulators could be an important biological control mechanism for appressorium development in *S. turcica*.

When developing the infection structure on the plant surface, pathogenic fungi secrete various carbohydrate‐active enzymes (CAZymes) to decompose the plant cell wall for further invasion. As a physical barrier that separates the challenging pathogen from the internal plant content, the plant cell wall is mainly composed of polysaccharides, specifically cellulose, hemicelluloses, and pectin. Fungal CAZymes include over 100 distinct glycosyl hydrolase (GH) families (Castillo et al., 2017). To date, the role of GH families in *S. turcica* during maize infection remains unknown.

Once the pathogenic fungus breaks down the cell wall, effector proteins become crucial for plant–microbe interactions. Effector factor prediction and analysis based on genome and transcriptome data are routinely reported in the plant fungal pathogenicity field (Jones et al., 2018; Lanver et al., 2017; Lo Presti et al., 2015). However, there is a dearth of information on essential effectors of *S. turcica*, although we have recently identified several candidate effectors encoded by the *S. turcica* genome (Wang et al., 2021). Identifying essential effectors and exploring their roles is another step toward understanding *S. turcica* pathogenicity.

Sequencing of the *S. turcica* genome has enabled functional annotation and characterization of development‐associated genes, CAZymes, and candidate effectors of this pathogen (Cao et al., 2020; Condon et al., 2013; Human et al., 2020; Ohm et al., 2012). The AvrHt1 gene was mapped to a hybrid PKS‐NRPS gene #179218 in the Et28A‐v1.0 genome (Mideros et al., 2018). Several signalling pathways regulating cell development and pathogenicity in *S. turcica* have been reported in the last decade (Gong et al., 2017; Gu et al., 2014; Shen et al., 2013; Zeng et al., 2020). Septins have been found to be required for conidia formation and virulence in the closely related maize pathogen *Cochliobolus heterostrophus* (Zhang et al., 2020).

A recent RNA sequencing (RNA‐Seq) study identified a number of common and novel putative effectors that expand the understanding of the *S. turcica*–maize interaction (Human et al., 2020). Multiple important quantitative trait loci associated with resistance to foliar maize diseases, including northern corn leaf blight, have been identified in the maize genome (Landi et al., 2005; Lopez‐Zuniga et al., 2019; Pozar et al., 2009; Yang et al., 2017). Importantly, the maize gene *Htn1* that confers resistance to northern corn leaf blight has been identified recently. However, knowledge about essential genes contributing to the pathogenesis of *S. turcica* at the early infection stages is still lacking.

In the current study, we aimed to identify novel essential genes required for *S. turcica* pathogenicity. To do this, we developed an in vitro assay to synchronize appressorium formation by the fungus on an artificial cellophane film. We then performed systematic analysis of the global transcriptional patterns during appressorium formation. Overall, the analysis revealed three different strategies that largely contribute to *S. turcica* pathogenicity, namely, cytoskeleton regulation, GH‐mediated cell wall degradation, and effector protein secretion. The expression of these essential factors was generally coupled to appressorium formation independent of host cell invasion. We showed that the cytoskeleton protein Slm2 is required for *S. turcica* pathogenicity and may control the morphological transition during appressorium maturation. In addition, we identified three novel appressorium‐coupled GH family proteins in *S. turcica* (namely, the GH domain‐containing proteins GH12, GH28, and GH74) that contribute to host cell wall degradation. Finally, we identified an appressorium‐coupled effector (StACE1) by systematic screening. The protein induces cell death of *Nicotiana benthamiana* and is required for maize infection. This work provides a critical foundation for further dissection of the roles of *S. turcica* factors in fungal interactions with the host plant during infection.

## 2 RESULTS

### 2.1 Genomic screening identified multiple factors that contribute to *S. turcica* pathogenicity

We have previously shown that the plant fungal pathogen *S. turcica* has evolved the capacity to breach the intact cuticle of the plant host by elaborating a specialized structure called the appressorium (Gu et al., 2014; Zhang et al., 2012). Expression of pathogenicity‐related genes is often coupled to appressorium development (Gu et al., 2014; Zeng et al., 2020; Zhang et al., 2012). We therefore performed transcriptome analysis of *S. turcica* during appressorium development to identify crucial genes that may play essential roles in fungal pathogenicity.

**FIGURE 1:**
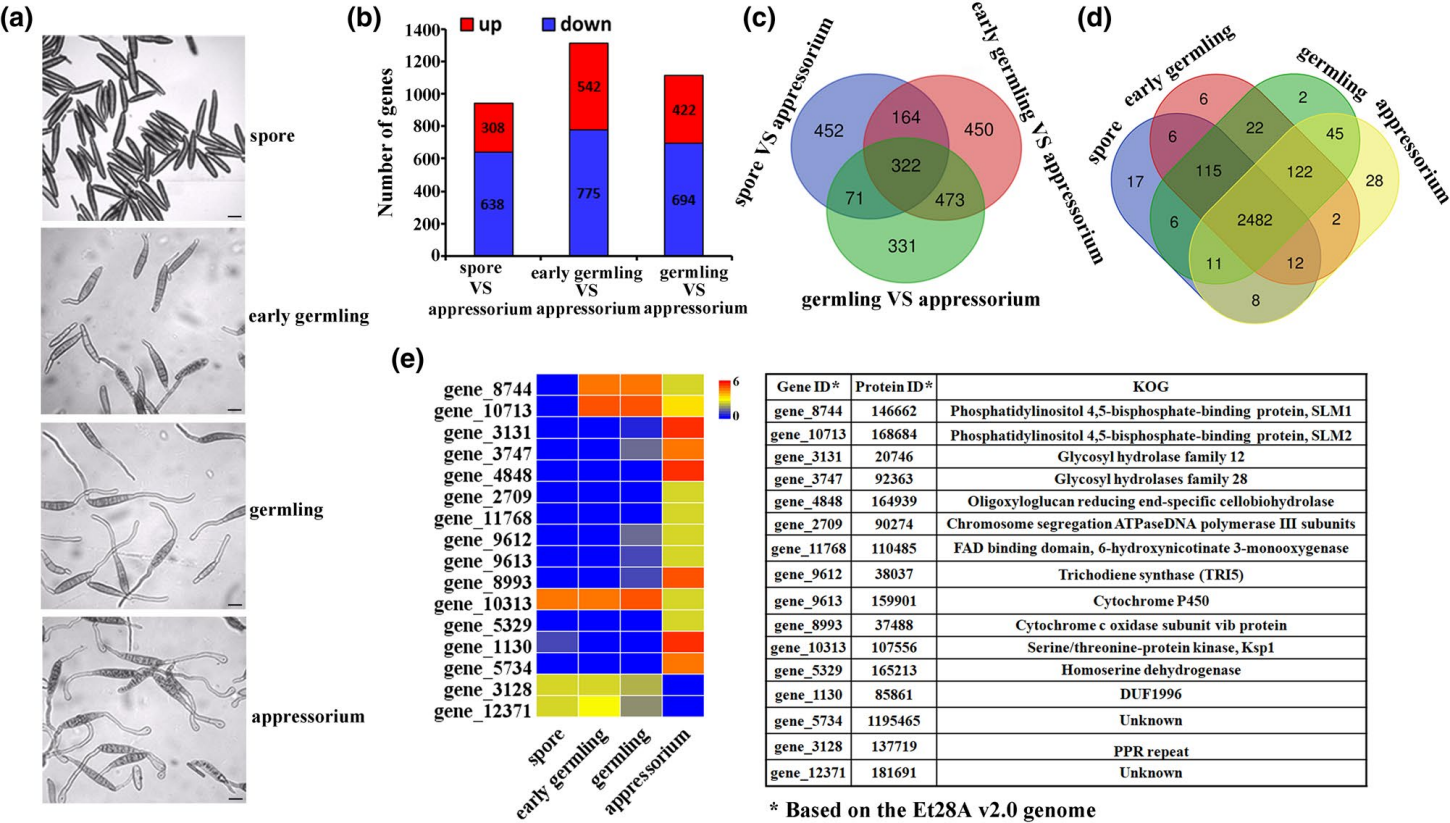
Transcriptomic profiling of fungal cells during appressorium development reveals essential candidate factors of *Setosphaeria turcica* pathogenicity. (a) Micrographs showing fungal morphology during appressorium development at synchronized time points (spore, 0 h; early germling, 3 h; germling, 6 h; and appressorium, 12 h) probed by RNA sequencing analysis (scale bar, 10 µm). Representative images are shown. (b) Differentially expressed gene (DEG) statistics for the indicated developmental stages. (c, d) Venn diagram of the numbers of shared and specific DEGs for the indicated developmental stages. (e) Heatmap expression of selected DEGs during the indicated developmental stages. The colour changes from red to yellow to blue in descending order of expression. Gene annotation is shown in the table

To ascertain the accuracy of the expression data, we first established an in vitro assay for synchronous induction of appressorium formation on an artificial cellophane film. We then used this system to identify genes showing specific expression patterns during appressorium formation by *S. turcica*. We selected specific analysis time points to target discrete developmental stages of appressorium morphogenesis, based on microscopic observation (Figure 1a). Accordingly, we constructed RNA‐Seq libraries of *S. turcica* wild‐type (WT) strain 01‐23 from conidia (0 h postinfection [hpi]), early germlings (3 hpi), germlings (6 hpi), and appressoria (12 hpi), which we then sequenced using an Illumina high‐throughput sequencing platform to obtain the specific transcriptome profiles. By comparing with the appressorium data, we identified significant differentially expressed genes (DEGs) that were either up‐regulated or down‐regulated in the spore, early germling, and germling (Figure 1b). A combined set of 2263 DEGs, including 322 shared genes, is presented in Figure 1c. We then analysed the four sets of DEGs shared by the different time points of appressorium formation (Figure 1b,c) to identify commonalities and specific differences. We used reads per kilobase per million mapped read (RPKM) values to determine gene expression levels and identified DEGs as described in Methods (Section 4.5). We identified 115 DEGs shared by samples from three stages (spore, early germling, germling) and 28 appressorium‐specific DEGs (Figure 1d).

As shown by a heatmap and annotation analysis (Figure 1e), we listed 16 out of 28 appressorium‐specific DEGs (Figure 1e), none of which had been characterized prior to the current study. We named the genes according to their homologs, if known, in other species (sequenced genomes) and in the EuKaryotic Orthologous Groups (KOG) database (Figure 1e). We thus identified genes for two cytoskeleton regulators (*SLM1* and *SLM2*), three GH proteins (*GH12*, *GH28*, and *GH74*), DNA mismatch repair protein 2 (*MSH2*), trichodiene synthase (*TRI5*), cytochromes P450, and several genes of unknown function, whose expression patterns indicated that they might be candidate factors essential for *S. turcica* pathogenicity (Figure 1e).

The data suggested that the identified novel genes might play conserved roles in *S. turcica* pathogenicity, as illustrated by the heatmap of DEG expression. Together, the results of the expression analysis of major shared DEGs and the identification of appressorium‐specific DEGs indicated that a large number of genes are probably related to appressorium development and pathogenicity. The heatmap of 16 appressorium‐specific DEGs reflected three different expression patterns: (a) expression increase specific to the appressorium time point, observed, for example for *GH12*, *GH28*, and *GH74*, among others; (b) expression increase at the germling time point and expression decrease at the appressorium time point, for example for *SLM1* and *SLM2*, and others; and (c) expression decrease specific to the appressorium time point. We selected three sets of genes exhibiting these different expression patterns for further analysis in an effort to relate the observed expression patterns to gene function.

### 2.2 Cytoskeleton regulator Slm2 is essential for hypha and conidium development

We were specifically interested in identifying gene expression patterns during appressorium development that were suggestive of physiological or signalling pathways important for cellular morphogenesis. *SLM1* and *SLM2* attracted our attention because their expression at different developmental stages was compatible with their possible function in morphology regulation during polarized and isotropic growth.

**FIGURE 2:**
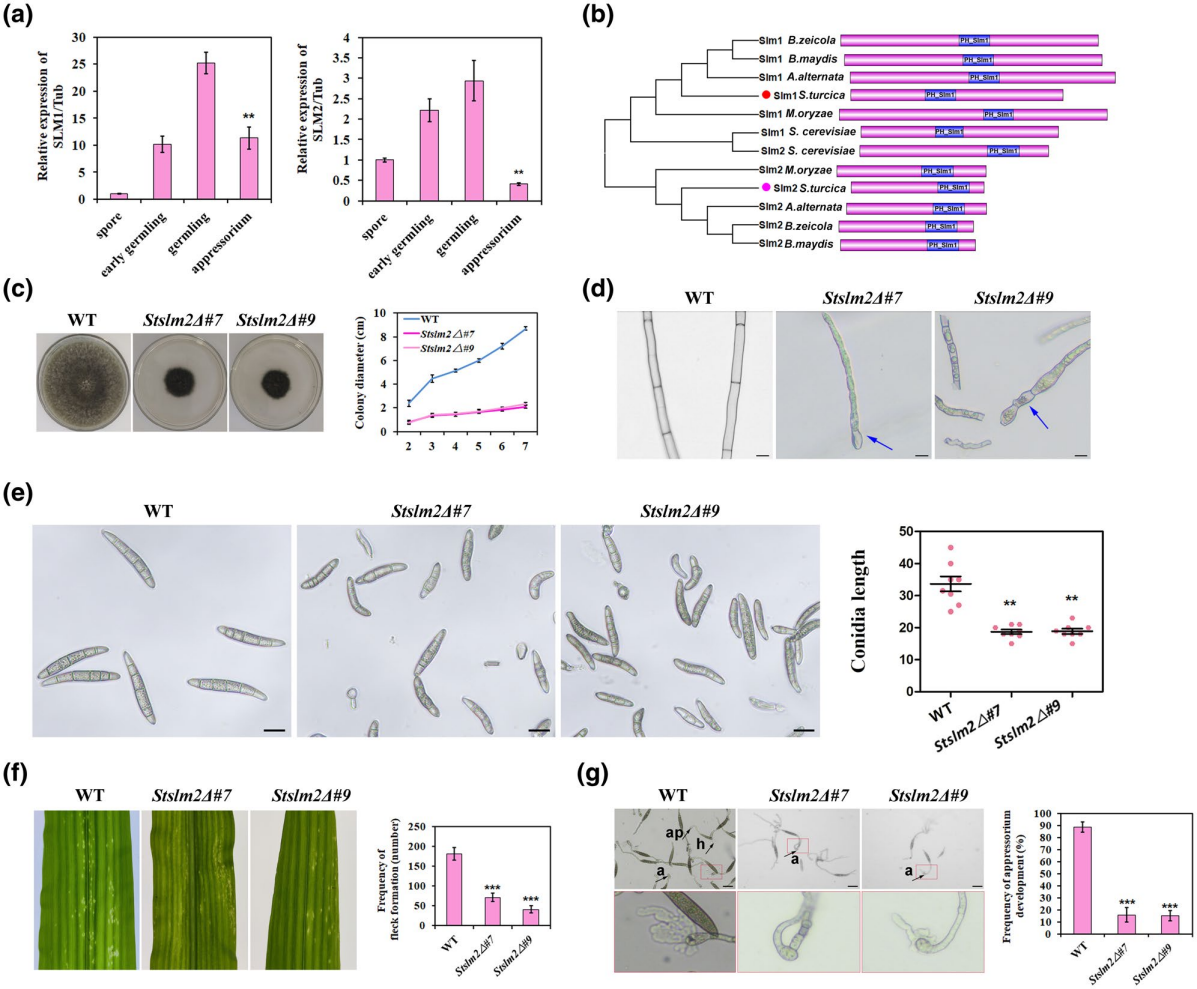
Cytoskeleton regulator Slm2 is essential for hypha and conidium development. (a) Reverse transcription quantitative PCR analysis of the relative transcript levels of *StSLM1* and *StSLM2* at the indicated time points during appressorium development (*n* = 3 independent replicates). (b) Conserved domains in Slm1 and Slm2 proteins of the indicated species. (c) Colony morphology and growth rate of the wild type (WT) and *Stslm2*Δ mutants on potato dextrose agar (PDA) at 25℃ in the dark. The colonies were photographed on day 7. The bar chart shows the growth rate of the indicated colonies in terms of diameter increase rate. (d) Micrographs showing mycelial morphology of the WT strain and *Stslm2*Δ mutants (scale bar, 10 µm). (e) Micrographs showing the morphology of WT and *Stslm2*Δ conidia. The scatter dot plot shows the length of WT and *Stslm2*Δ conidia. **p < 0.01 (spores observed = 100; scale bar, 10 µm). (f) Pathogenicity of *Stslm2*Δ mutants is reduced compared with that of the WT. WT and *Stslm2*Δ conidia were spread on the fifth leaf of maize B73 seedlings. Typical leaf symptoms were photographed and the number of lesions was scored at 4 days postinoculation (dpi). The bar chart shows the frequency of flecks formation on leaves (number/10 cm^2^) inoculated with the WT strain and *Stslm2*Δ mutants. ***p < 0.001 (*n* = 3 independent replicates). (g) Micrographs showing appressorium formation and maize infection by the WT strain and *Stslm2*Δ mutants. The conidial suspension was incubated on cellophane at 25℃ in the dark and the fungi were analysed 12 h postinoculation (hpi). a, appressorium; ap, appressorium pore; h, hypha. The bar chart shows the frequency of appressorium formation on cellophane. ***p < 0.001 (*n* = 3 independent replicates; spores observed = 100; scale bar, 10 µm)

We first confirmed the *SLM1* and *SLM2* expression patterns by reverse transcription quantitative PCR (RT‐qPCR). The gene expression was significantly up‐regulated during early germling (3 hpi) and germling (6 hpi) stages, and down‐regulated during the appressorium stage (12 hpi), with a pattern similar to that detected by RNA‐Seq (Figure 2a). Multiple‐sequence alignment with orthologous proteins from some model fungi revealed structural conservation (Figure 2b), indicating that Slm1 and Slm2 are evolutionarily conserved in fungal species. These cytoskeleton regulator proteins may be involved in germling and appressorium development, because conidial germling formation is a biological process similar to yeast budding, and appressorium development from the germ tube tip is similar to the process of isotropic growth from the bud tip (Oses‐Ruiz & Talbot, 2017).

To gain insight into the roles of Slm1 and Slm2 in fungal pathogenicity, we generated *SLM1* and *SLM2* knockout mutants of *S. turcica*. We obtained two stable transformants (*Stslm2*Δ#7 and *Stslm2*Δ#9) of the *slm2* deletion mutant derived from the WT strain 01‐23. However, we did not obtain an *slm1* mutant despite multiple trials, indicating that Slm1 might be essential for *S. turcica* viability. We therefore performed further functional analyses using the *Stslm2* deletion mutants.

The *Stslm2* deletion mutants exhibited a severe growth defect (Figure 2c) and their hyphal morphology was dramatically affected. Compared with the WT, the hyphal length‐to‐width ratio of the two *Stslm2* knockout mutants was generally reduced, and the cells were almost round (Figure 2d, arrows). The mutant conidia were significantly shorter than WT conidia (Figure 2e). These observations indicated that Slm2 may directly regulate cell morphology in *S. turcica*, similar to the function of the yeast Slm1/2 ortholog in actin polarization.

To assess the ability of *Stslm2Δ* mutants to infect maize and cause disease symptoms in maize, we inoculated the fifth leaf of maize seedlings with equal volumes of conidial suspensions of the WT and mutant fungi. Flecks appeared on the leaves inoculated with WT spores 4 days postinoculation (dpi). The number of flecks formed on the leaves inoculated with mutant spores (*Stslm2*Δ) were significantly lower than that formed by the WT strain (Figure 2f). To further analyse the appressorium formation and penetration ability of *Stslm2*Δ mutants, we compared strain pathogenicity by using in vitro penetration assay. Cellophane induced mutant conidial formation, consistent with the observations for leaf infection. However, appressorium formation and penetration by *Stslm2*Δ mutants were largely inhibited on cellophane compared with those of the WT strain (Figure 2g). These observations indicate that StSlm2 is required for appressorium development and invasive growth, both of which contribute to *S. turcica* pathogenicity.

### 2.3 Products of GH genes with appressorium‐coupled expression degrade the plant cell wall

Once pathogenic fungi land on the leaf of the host plant, they frequently secrete cell wall‐degrading enzymes to break down the cell wall. Our phylogenetic analysis indicated that orthologs of *S. turcica* GH proteins are widely present in fungi, including some important plant pathogens, such as *Alternaria alternata*, *Bipolaris maydis*, and *Magnaporthe oryzae* (Figure S1a). Sequence analysis revealed that *S. turcica* GH proteins have a 20 amino acid signal peptide and no transmembrane helices, implying that they are secreted. Furthermore, bioinformatics analysis suggested that these proteins possess the highly conserved GH domain and represent three GH families (GH12, GH28, and GH74) (Figure S1b). The predicted three‐dimensional structures of these proteins are shown in Figure S1c.

**FIGURE 3:**
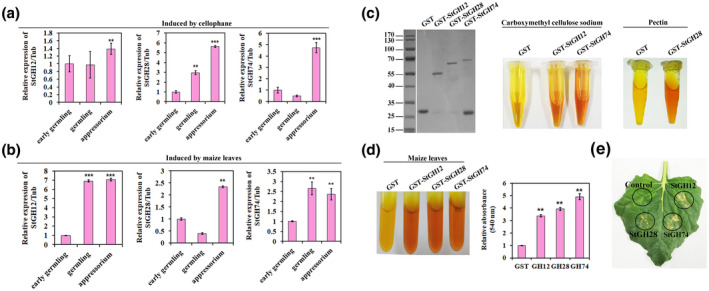
Appressorium‐coupled expression of GH genes and degradation of host plant cell wall. (a, b) Reverse transcription quantitative PCR analysis of the relative expression of the *StGH12, StGH28*, and *StGH74* genes induced by cellophane and maize leaves during the indicated developmental stages, ***p < 0.001 (*n* = 3 independent replicates). (c) Purification and activity of three GH proteins. SDS‐PAGE of the purified recombinant proteins. The gel was stained with Coomassie brilliant blue. The activity of the GH proteins was analysed by the DNS reducing sugar method, with sodium carboxymethyl cellulose and pectin as substrates. Reaction mixtures containing GH12, GH28, and GH74 turned red in the assay. GH12 and GH74 degrade cellulase, and GH28 degrades pectin. (d) Results of co‐incubation of purified recombinant glutathione *S*‐transferase (GST)‐tagged GHs with maize leaves in a reaction buffer at 24℃ for 1 h. The experiment indicated that the proteins degrade maize leaves. The bar chart shows the relative sample absorbance at 540 nm. **p < 0.01 (*n* = 3 independent replicates). (e) Plant leaf degradation by three GH proteins. For the experiment, 25 µl of GH suspensions were spread on tobacco leaves and incubated in artificial‐climate chambers under long‐day conditions at 25℃

In the transcriptome analysis, we observed that the expression of the three GH genes is coupled to appressorium formation. We confirmed this expression pattern by RT‐qPCR, as shown in Figure 3a. We also detected GH gene expression coupled to appressorium formation on maize leaf (Figure 3b).

To analyse the enzymatic functions of GH proteins in *S. turcica*, we performed a series of biochemical assays with purified, recombinant glutathione *S*‐transferase (GST)‐tagged GH proteins and the potential substrates sodium carboxymethyl cellulose and pectin. We incubated the GST and GST‐GH proteins in the presence of the substrates for 1 h at 24℃ and used the 3,5‐dinitrosalicylic acid (DNS) reducing sugar method to determine GH activity. The analysis indicated that the recombinant GH proteins were active (Figure 3c).

Next, to investigate whether appressorium‐specific GH proteins can degrade the plant cell wall, we treated plant leaves with the proteins. As shown in Figure 3d, the GH proteins degraded the cell wall of maize, based on the results of the DNS reducing sugar assay. To examine the cell wall‐degrading activity of GH proteins on plants other than maize, we infiltrated the leaves of *N. benthamiana* with the recombinant proteins. GH proteins caused significant cell wall degradation, while the GST control did not (Figure 3e).

### 2.4 Secreted *S. turcica* protein StACE1 is a novel effector that induces plant cell death

To establish successful colonization, *S. turcica*, like other fungal pathogens, secretes a large number of effectors during appressorium‐mediated infection. In the course of transcriptome analysis (Section 2.1), we identified a small protein that exhibited an appressorium‐coupled expression pattern, which we named appressorium‐coupled effector 1 (StACE1).

**FIGURE 4:**
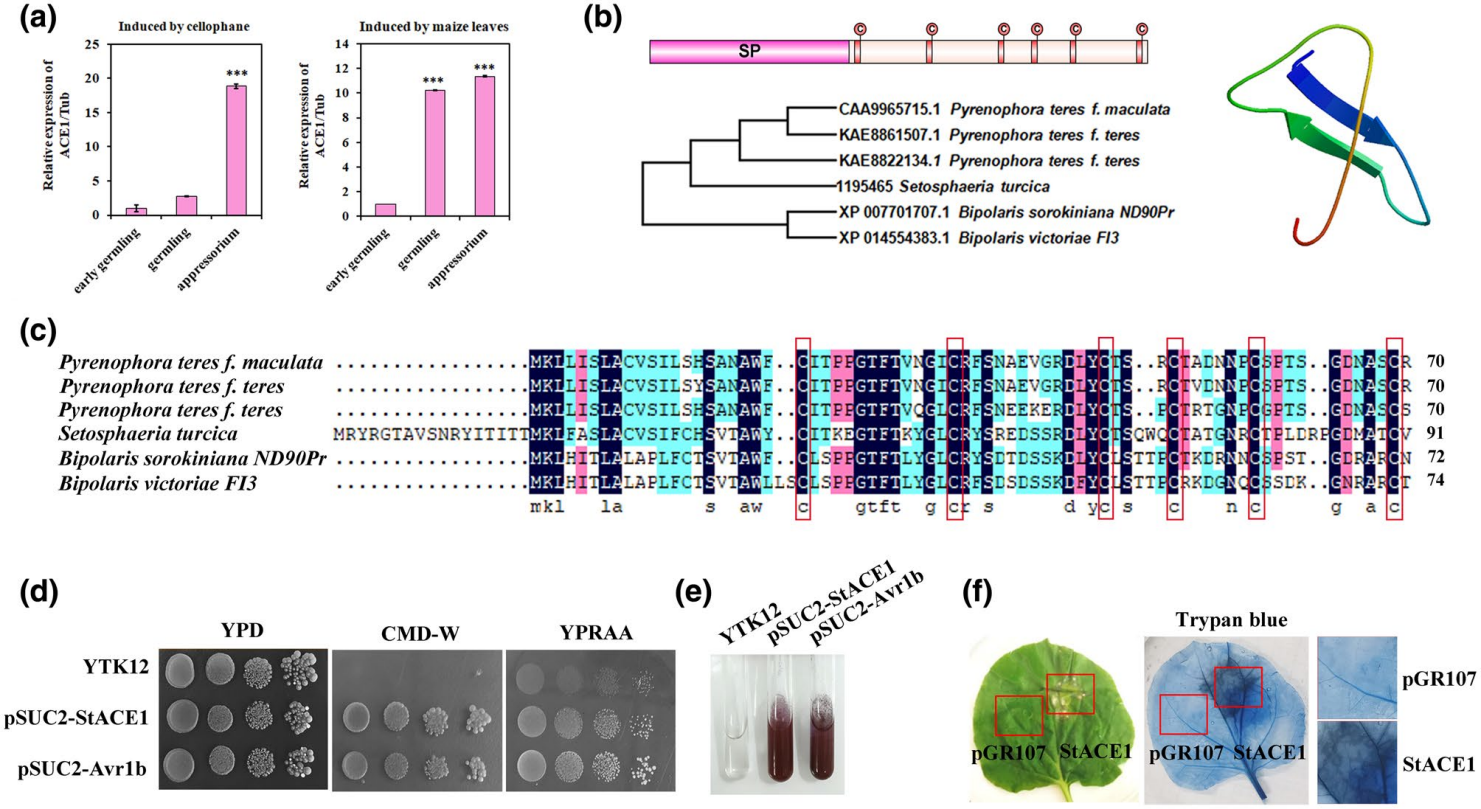
Secreted *Setosphaeria turcica* protein StACE1 is a novel effector that induces plant cell death. (a) Relative *StACE1* transcript accumulation, determined by reverse transcription quantitative PCR, induced by cellophane or maize leaves at various time points during appressorium development. Relative transcript levels were calculated using the comparative *C*_t_ method. Data were normalized using *S. turcica* *β‐tubulin* transcript levels. Values (bars) are the means of three independent trials, with the error (±*SD*) shown. ***p < 0.001 (*n* = 3 independent replicates). (b) StACE1 structure analysis. Signal peptide (sp) and cysteine residues are indicated. (c) Phylogenetic analysis and multiple amino acid sequence alignment of StACE1 and its orthologs. (d) Functional evaluation of the StACE1 signal peptide. The secretion activity of the StACE1 signal peptide was confirmed using a yeast secretion system. Strain YTK12, harbouring pSUC2‐StACE1, pSUC2‐Avr1b, or an empty vector, was cultured on YPD, CMD−W (Dropout−Trp), and YPRAA medium. In the system, the yeast only grows on active invertase secretion. Invertase activity was detected based on the reduction of 2,3,5‐triphenyltetrazolium chloride to the insoluble, red‐coloured 1,3,5‐triphenylformazan. (e) StACE1 induces cell death in *Nicotiana benthamiana*. Leaves of *N. benthamiana* were agroinfiltrated with *Agrobacterium tumefaciens* GV3101 containing an empty vector pGR107‐35s‐eGFP or pGR107‐35s‐StACE1‐ΔSP. Cell death was visualized 5 days postinoculation (dpi) after agroinfiltration, by trypan blue staining

We first analysed the expression of *StACE1* during different stages of appressorium development induced by cellophane or maize leaves. RT‐qPCR analysis suggested that when artificial cellophane or maize leaves were inoculated with *S. turcica* spore suspension, *StACE1* transcript levels increased rapidly, with a maximum increase of approximately 18‐fold and 11‐fold at 12 hpi, respectively (Figure 4a). This expression pattern was in agreement with the pattern detected by RNA‐Seq (Figure 1e).

Next, we used bioinformatics to explore the biological roles of StACE1. The *StACE1* open reading frame consists of 276 bp that encodes a small protein of 91 amino acids, with a predicted N‐terminal signal peptide (sp) (amino acids 1–36), suggesting that StACE1 is secreted. Furthermore, StACE1 has six well‐conserved cysteine residues (C39, C52, C65, C71, C78, and C90) (Figure 4b,c). In addition, this protein is only present in few fungal species, including *Pyrenophora teres*, *Bipolaris sorokiniana*, and *Bipolaris victoriae* (Figure 4b,c).

We used a yeast secretion system to verify the function of the predicted signal peptide of StACE1. We introduced DNA sequences encoding the StACE1 candidate signal peptide and that for Avr1bSP (the signal peptide of Avr1b) into the plasmid pSUC2, and then used the constructs to transform the yeast strain YTK12, which lacks a secreted invertase. We streaked the transformants onto CMD−W (SD−Trp) and YPRAA media, which only support the growth of yeast with a functional secreted invertase (Figure 4d). We then detected the enzymatic activity of secreted invertase based on the reduction of 2,3,5‐triphenyltetrazolium chloride (TTC) to the red‐coloured 1,3,5‐triphenylformazan. Reaction mixtures containing transformants harbouring *StACE1SP* and *Avr1bSP* genes turned red, while the YTK12 strain did not (Figure 4e). These observations suggested that StACE1 contains a signal peptide and is a secreted protein.

To characterize the function of StACE1 during infection, we transiently expressed *StACE1* in *N. benthamiana* by agroinfiltration. We used pGR107‐based expression vectors to express full‐length StACE1 or StACE1‐ΔSP (lacking the signal peptide) in *N. benthamiana*. We observed that StACE1 or StACE1‐ΔSP, but not the empty vector, induced pronounced leaf cell death, suggesting that StACE1 specifically induced plant cell death (Figure 4f). Together, these observations imply that StACE1 is critical for the plant cell death‐inducing activity of *S. turcica*.

### 2.5 StACE1 is not required for appressorium development but is essential for virulence

**FIGURE 5:**
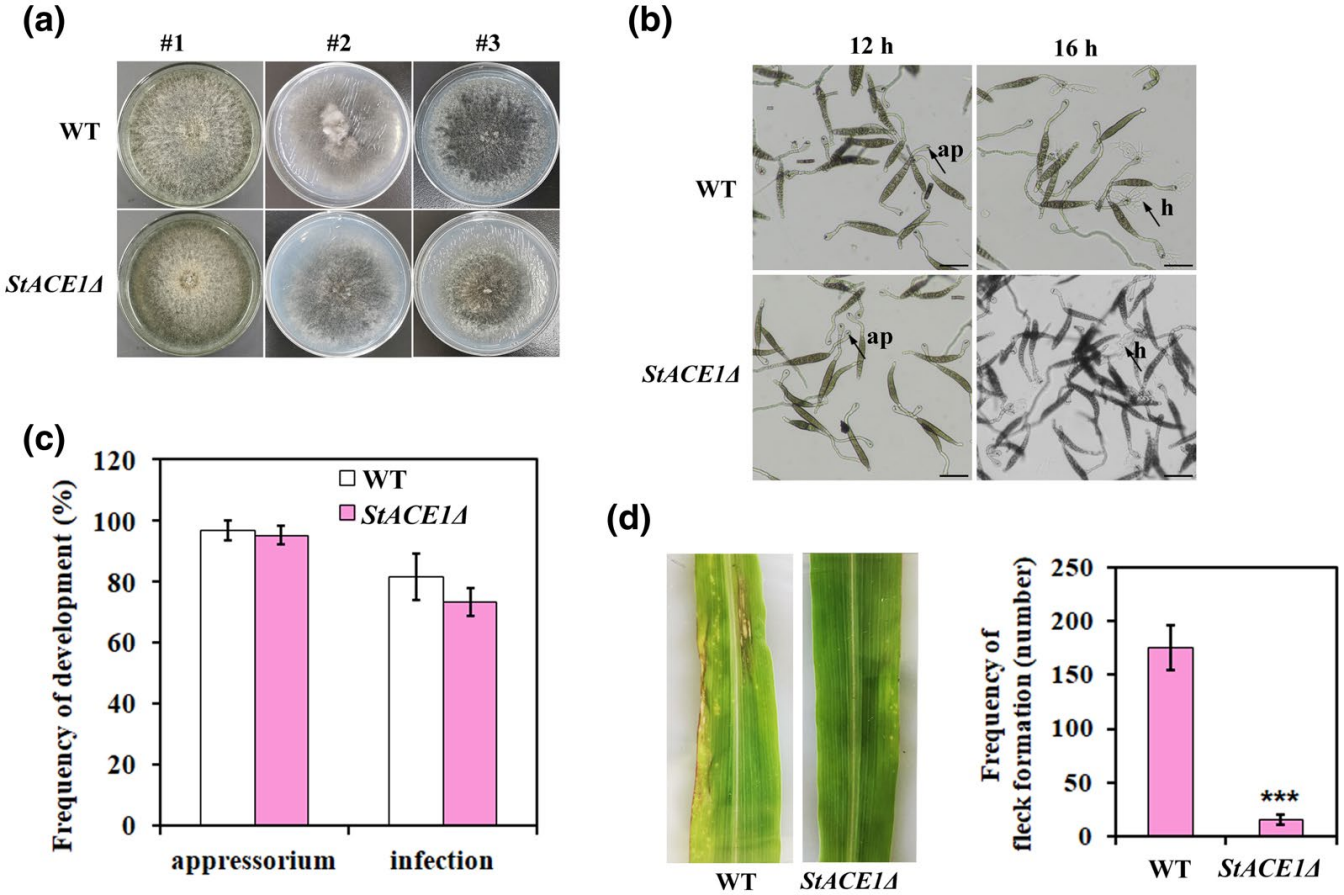
StACE1 is not required for appressorium development but is essential for virulence. (a) Phenotype of the wild‐type (WT) strain and *StACE1*Δ mutant grown on potato dextrose agar (PDA) at 25℃ in the dark. Cellophane membrane penetration was not affected in the *StACE1*Δ mutant. #1, without cellophane; #2 and #3, conidial and hyphal growth after cellophane penetration. (b) Micrographs of *StACE1*Δ mutant showing appressorium formation at 12 h postinoculation (hpi) and cellophane penetration at 16 hpi. ap, appressorium pore; h, hypha (scale bar, 10 µm). (c) Bar chart showing the frequency of appressorium formation and penetration by the WT and *StACE1*Δ strains on cellophane. ***p < 0.001 (*n* = 3 independent replicates; spores observed = 100). (d) WT and *StACE1*Δ conidia were spread on the fifth leaf of maize B73 seedlings, and typical leaf disease symptoms were photographed and the lesion number was scored at 4 days postinoculation (dpi). The bar chart shows the frequency of flecks formation on leaf (number/10 cm^2^) inoculated with the WT and *StACE1*Δ strains. ***p < 0.001 (*n* = 3 independent replicates)

StACE1 is a secreted protein and the *StACE1* gene is highly expressed during the appressorium stage (12 hpi) (Figure 4a), indicating that StACE1 may be involved in plant infection by *S. turcica*. To explore the biological roles of StACE1 in *S. turcica*, we generated *StACE1* deletion mutant by gene replacement with the hygromycin B (*hph*) resistance cassette. We did not observe any significant differences between the *StACE1*Δ mutants and the WT strain 01‐23 in terms of colony morphology, growth rate, appressorium development, and penetration (Figure 5a–c). However, the virulence of the *StACE1*Δ mutants was significantly reduced, and the mutant produced fewer and much smaller lesions on maize leaf than the WT strain 01‐23 at 24 hpi (Figure 5d). These observations demonstrated that StACE1 plays an important role in the virulence of *S. turcica*.

## 3 DISCUSSION

The fungal pathogen *S. turcica* is of great economic importance because it threatens the production of maize already growing in the field (Van Inghelandt et al., 2012). To cause maize foliar disease, *S. turcica* forms the appressorium, a specialized infection structure. Understanding the *S. turcica* germination process and appressorium development, as well as related biological events, could lead to improved disease control, as these processes are one of the earliest stages of fungal preparation for plant infection. Our laboratory has a long‐time interest in and focuses on the molecular control of *S. turcica* pathogenicity and fungicide development (Cao et al., 2020; Huo et al., 2018). Fieldwork observations suggest that inhibition of appressorium development is an efficient strategy against *S. turcica*‐mediated maize foliar disease (Zeng et al., 2020). We here investigated the gene expression patterns during appressorium development and show that the phytopathogen *S*. *turcica* is primed in advance with multiple strategies for maize infection.

We designed the current study to identify genes essential for *S. turcica* pathogenicity, especially those whose expression is coupled to appressorium development. Appressorium formation is regulated by reorientation of the polarity axis. Accordingly, we here demonstrated that the cytoskeleton regulator Slm2 is essential for *S. turcica* cell morphology and therefore contributes to maize infection. In yeast, Slm1 and Slm2 regulate processes from polarized hyphal growth to isotropic expansion, cell budding, and bud growth (Audhya et al., 2004; Fadri et al., 2005). Cytoskeleton construction during polarization and isotropic expansion in spore germling and appressorium formation is similar, as in the process of yeast budding and bud growth (Oses‐Ruiz & Talbot, 2017). Furthermore, appressorium formation involves a switch from polarized hyphal growth to isotropic expansion of the germ tube tip. According to previous studies in the yeast *Saccharomyces cerevisiae*, Slm1/Slm2, acting downstream of PI4,5P_2_ and the TORC2 kinase pathway, is required for actin cytoskeleton polarization (Audhya et al., 2004). Target‐of‐rapamycin proteins (TORs) are Ser/Thr kinases with a central role in cell growth control and thus are promising targets for fungicide development. This study revealed that the expression patterns of Slm1 and Slm2 in *S. turcica* are compatible with their functions in actin cytoskeleton polarization and morphological changes during spore germling and appressorium formation. We showed that Slm2 plays a specific role in *S. turcica* pathogenicity as a cytoskeleton regulator, suggesting that it could be targeted for the development of novel fungicidal agents.

**FIGURE 6:**
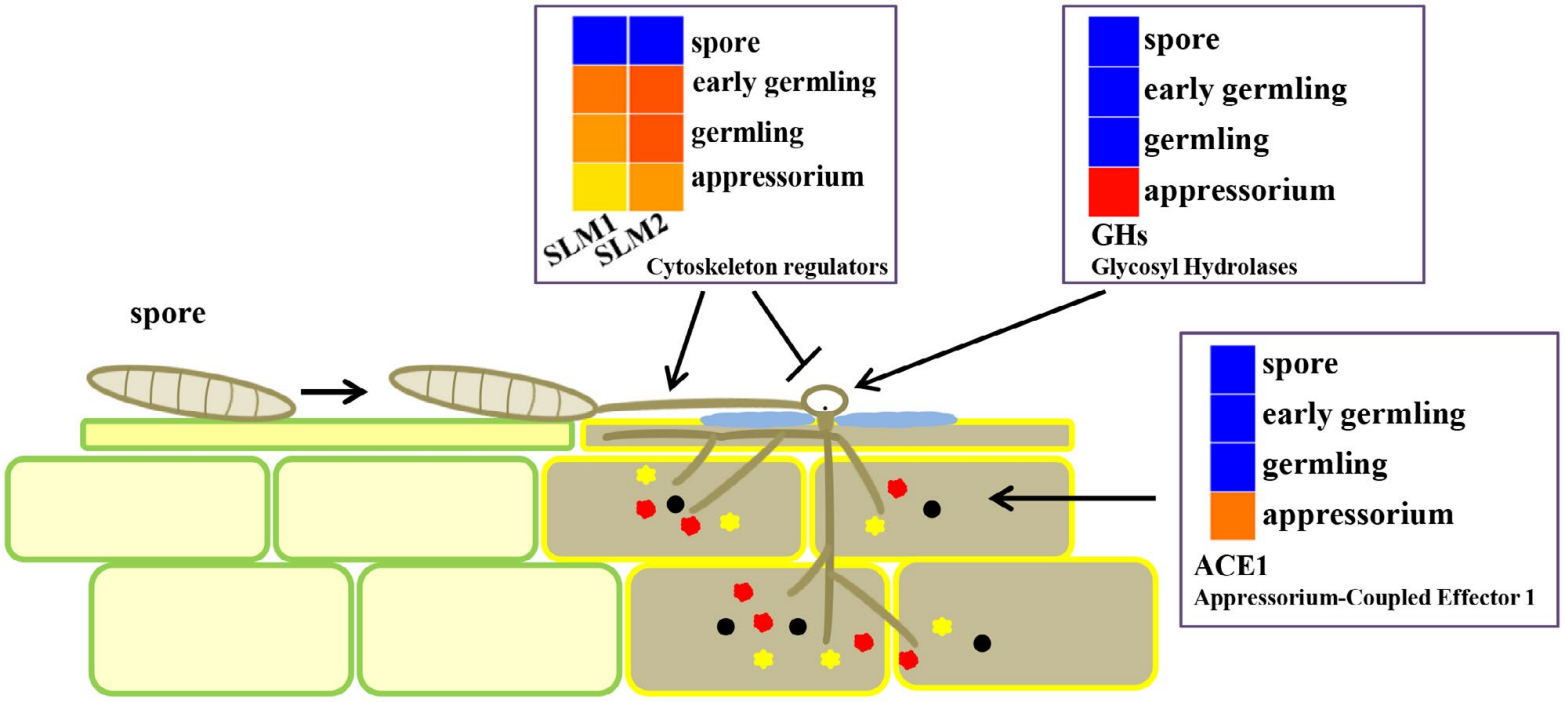
Working model of the newly identified factors contributing to *Setosphaeria turcica* pathogenicity at the early stages of maize infection. A conidium germinates and forms an appressorium that is coupled to differential expression of virulence factors, indicating a coordinated stage‐specific expression of diverse pathogenesis mechanisms. Cytoskeleton regulators Slm1 and Slm2 are involved in the appressorium formation. The heatmap shows up‐regulation of *SLM1* and *SLM2* expression at the early and middle infection stages, and a significant down‐regulation during the appressorium stage. Appressorium‐coupled GH proteins accumulate in the mature appressorium and degrade the cell wall of the host plant. The expression of StACE1, a novel specific effector of *S. turcica*, is significantly increased at the mature appressorium stage. StACE1 induces cell death and plays an important role in virulence (blue for low, red for high expression level)

In the current study, we showed that the phytopathogen *S. turcica* has evolved multiple factors required for appressorium formation or virulence, which contribute to fungal pathogenicity and maize infection (Figure 6). We demonstrated that the expression of Slm1/2 is regulated to coincide with spore germination and appressorium formation, and is required for effective appressorium‐mediated infection. A coordinated stage‐specific expression is presented of diverse pathogenesis mechanisms, in that the expression of pathogenicity‐related factors accompanies appressorium development (Figure 6). GH enzymes degrade the plant cell wall, thus destroying the first line of plant defence. Following entry into the host leaf, *S. turcica* undergoes further developmental switching to form specialized invasive hyphae, which secrete effector proteins and counter host cell defences. The plant cell wall is a physical barrier that the phytopathogenic fungi must overcome by producing an array of cell wall‐degrading enzymes, which allows them to invade host tissues by degrading cell wall components (Quoc & Chau, 2017).

We showed here that appressorium‐coupled effectors of *S. turcica* triggered plant cell death on transient expression in *N. benthamiana*. Furthermore, deletion of *StACE1* significantly reduced fungal virulence. These observations suggest that StACE1 is important for *S. turcica* virulence and that it might be recognized by plants to trigger plant defence responses. Pathogenic fungus effectors function in various ways: they can shield the pathogen, inactivate plant enzymes or toxic compounds that are harmful to the pathogen, prevent the elicitation of plant immune responses, or alter the physiology of the infected plant to support the growth and development of the pathogen (Jones et al., 2018; Lanver et al., 2017; Lo Presti et al., 2015). The molecular details of StACE1 involvement in *S. turcica*–maize interaction should be explored in future studies.

RNA‐Seq is a powerful tool for visualizing transcriptome complexity, enabling genome‐wide identification of coding sequences with possible essential roles related to the specific time point during plant infection. This analysis is important because it constitutes the first step toward functional characterization of genes and offers opportunities to examine the relationship between the host and pathogen to identify novel strategies that could be used for therapeutic and prophylactic interventions (Soanes et al., 2012; Wang et al., 2009).

The time‐course transcriptome analysis presented herein revealed that genes encoding TRI5, cytochromes P450, and a FAD protein, among others, were highly expressed at the appressorium stage (Figure 1e). We confirmed the expression of these genes in conjunction with appressorium induction on cellophane and maize leaf (Figure S2). The relationship between transporters, cytochromes P450, transcription factors, and pathogen virulence is widely known. For example, cytochromes P450 are involved in detoxification of host toxins and allow pathogenic fungi to grow under different conditions (Cresnar & Petric, 2011). Therefore, these highly expressed genes may play conserved roles in fungal pathogenesis, regardless of the infection stage and fungal species. The factors identified in this study present a coordinated stage‐specific expression of diverse pathogenesis mechanisms. We plan to explore the detailed mechanisms of fungal pathogenicity in the future, using the RNA‐Seq database generated in the current study.

Many questions regarding the molecular details of fungal pathogenicity should be answered to identify appropriate strategies to control *S. turcica* infection in maize and enhance the disease resistance of maize against the attack of fungal pathogens. In the current study, we showed that some essential factors are actually expressed in conjunction with appressorium formation, which is independent of *S. turcica*–maize encounters: first, the cytoskeleton regulatory proteins control cell morphogenesis during appressorium development; second, appressorium‐coupled expression of GH genes allows for the degradation of the cell wall of the host plant; and finally, specific effectors induce host cell death. Identification of *S. turcica* genes whose expression is appressorium development‐specific and detailed information about their function in fungal virulence will provide an improved understanding of *S. turcica* behaviour during infection at a molecular level.

## 4 EXPERIMENTAL PROCEDURES

### 4.1 Strains, plant material, and culture conditions

The *S. turcica* strain 01‐23 (the WT strain) used in the current study was isolated from northern corn leaf blight samples of maize leaf in Liaoning Province, China. It has been deposited in the China General Microbiological Culture Collection Centre (no. 9857). The WT strain and strain 01‐23–derived *Stslm2* (*Stslm2*Δ#7 and *Stslm2*Δ#9) and *StACE1* knockout mutants were grown on potato dextrose agar (PDA; 20% potato, 2% glucose, and 1.5% agar) at 25℃. *S. turcica* growth, storage, and transformation were all performed using standard procedures, as described previously (Zeng et al., 2020).

Maize inbred line B73 was used as the susceptible host for *S. turcica* infection. It was grown in artificial‐climate chambers under long‐day conditions (16 h light/8 h dark). Maize growth and infection were as described previously (Zeng et al., 2020). The yeast YTK12 strain was routinely grown on YPD medium (1% yeast, 2% peptone, 2% glucose, and 2% agar) at 30℃, and the yeast genetic manipulations followed standard procedures (Li et al., 2020a, 2020b; Liu et al., 2020). *Agrobacterium tumefaciens* GV3101 was used for *Agrobacterium*‐mediated transient gene expression in plant leaves. *Escherichia coli* DH5α and BL21 were used for plasmid amplification and protein purification. *N. benthamiana* plants were grown in artificial‐climate chambers at 23℃ under a 16 h/8 h dark cycle. The protocols related to *N. benthamiana* manipulation were all performed using standard procedures, as described previously (Zheng et al., 2019).

### 4.2 Generation of *Stslm2Δ* and *StACE1Δ* mutants

Constructs used to generate *Stslm2*Δ and *StACE1*Δ mutants were derived from the pBS‐bar/pBS‐hph vector. Targeted replacement of *SLM2* and *ACE1* was performed by using bialaphos (*bar*) and *hph* selectable markers, respectively. The *slm2:bar* constructs were introduced into *S. turcica*, and transformants were selected on PDA containing 200 µg/ml glufosinate. The *ACE1:hph* transformants were selected on PDA containing 75 µg/ml hygromycin B. Vector construction for allelic replacement to generate *Stslm2*Δ and *StACE1*Δ mutants followed standard procedures, as described previously (Gu et al., 2014; Nakayashiki et al., 2005; Zeng et al., 2020; Zhang et al., 2012).

### 4.3 Appressorium formation and plant infection assays

Appressorium development was induced in vitro on an artificial cellophane film using an adaptation of a previously described method (Ma et al., 2018; Zeng et al., 2020). For the experiment, 25 µl of conidial suspension (5 × 10^4^/ml) was placed on an artificial cellophane (Solarbio) and incubated at 24℃ in the dark. Mycelium penetration and appressorium formation were observed at the indicated time points using a microscope.

Maize lesions and appressorium development on leaves were observed using a plant infection assay, as described previously, with minor modifications (Ma et al., 2018; Zeng et al., 2020). Briefly, 100 µl of conidial suspensions (10^7^ conidia/ml in 0.25% gelatin solution) of each strain were spread on maize leaves of inbred B73 line in artificial‐climate chambers under long‐day conditions at 25℃. Flecks were counted 4 dpi on 5 cm‐long diseased maize blade sections taken from the leaves at the point of inoculation. Data were obtained from triple independent replicates.

### 4.4 RNA extraction and library preparation for RNA‐Seq

Plant samples were ground into powder in liquid nitrogen, total RNA was extracted, and mRNA was isolated. After quality control, poly(A)‐tailed transcripts were isolated by oligo(dT) selection using streptavidin‐coated magnetic beads and randomly fragmented by Mg^2+^ ion treatment (Choi et al., 2015). Then, cDNA was synthesized using random hexamer primers and mRNA as a template. The resultant products were connected to adapters. This was followed by size selection and PCR amplification. The constructed library was analysed using Illumina NovaSeq 6000 sequencing platform (Biomics). The RNA‐Seq data have been deposited under the accession number SUB7702464 on the NCBI server (http://www.ncbi.nlm.nih.gov/sra).

### 4.5 RNA‐Seq experiment and analysis

Adapter sequences and low‐quality reads were filtered out from the raw data, and paired reads were selected for further analysis. The clean reads were then aligned to the reference genome of *S. turcica* strain Et28A v. 2.0 (Condon et al., 2013; Ohm et al., 2012). Fragments per kilobase of transcript per million mapped reads (FPKM) values were calculated using Cufflinks software, as an indicator of the level of gene expression. The FPKM values were applied to two sample libraries at a time to determine log_2_ ratio of difference in expression (spore, early germling, germling, and appressorium). DEGs were filtered and corrected at a false discovery rate (FDR) ≤ 0.01. Genes were annotated by BLAST‐searching against the local nr database (http://ftp‐private.ncbi.nlm.nih.gov). Functional genes were identified using the Gene Ontology (http://www.geneontology.org/), KEGG (http://www.genome.jp/kegg/), and Pfam (http://pfam.xfam.org) databases.

### 4.6 RNA extraction and RT‐qPCR analysis

Total RNA was extracted using Fungal RNA Kit (Omega). It was then reverse‐transcribed to cDNA using M‐MLV reverse transcriptase (ABclonal), according to the manufacturer's instructions. Gene expression was determined by qPCR with SYBR Green I (Takara) using CFX96 Touch System (Bio‐Rad), following a previously described protocol (Gu et al., 2014; Zeng et al., 2020). To calculate the expression of target genes, data were analysed using the 2^−ΔΔ^ *^C^* ^t^ method, with the expression of the *β‐tubulin* gene as an internal control, as described previously (Bustin et al., 2009; Zeng et al., 2020).

### 4.7 Bioinformatics analysis

Reference sequences of DEGs were retrieved from the NCBI GenBank database. Sequence alignment was performed using the BLASTP program at the *S. turcica* genome database (https://mycocosm.jgi.doe.gov/pages/blast‐query.jsf?db=Settu3). SignalP v. 4.0 (http://www.cbs.dtu.dk/services/SignalP/) was used for signal peptide prediction; TMHMM 2.0 (http://www.cbs.dtu.dk/services/TMHMM/) was used for the prediction of transmembrane helices. The SWISS‐MODEL database was used for protein homology modelling (https://swissmodel.expasy.org/interactive), and the generated PDB file was edited using PYMOL software. Amino acid sequence alignments were generated in Muscle. The phylogenetic tree was constructed in MEGA 5.0 using the maximum‐likelihood method. Sequence motifs were identified and analysed using the online web server provided as part of the MEME suite (Yang et al., 2018).

### 4.8 Protein purification and DNS reducing sugar assay

GST‐StGH12, GST‐StGH28, and GST‐StGH74 were expressed in *E. coli* BL21 and purified by affinity chromatography using glutathione–sepharose beads (GE Healthcare) according to the manufacturer's recommendations, as described previously (Zeng et al., 2020).

DNS solution contained 6.3 g DNS, 262 ml of 2 M NaOH, 185 g potassium sodium tartrate, 5 g crystallized phenol, and 5 g sodium sulphite in 1 L. The solution was kept in the dark for at least 1 week before use (Zhang et al., 2019). The reducing sugar activity of proteins was assayed as described previously. Briefly, GST‐GH proteins were mixed with a reaction solution that contained sodium carboxymethyl cellulose or pectin and 50 mM Tris‐HCl, pH 8.0, and kept at 24℃ for 1 h. Then, 1 ml of DNS solution was added and the samples were incubated at 100℃ for 10 min. After cooling to room temperature, 200 µl of the mixture was transferred to a 96‐well plate, and sample absorbance at 540 nm was determined.

### 4.9 Yeast secretion assay

DNA sequences encoding the signal peptides of StACE1 and Avr1b were inserted between the *Eco*RI and *Xho*I sites of pSUC2 vector and expressed as SP‐SUC2 fusion protein. Yeast strain YTK12 was then transformed with the pSUC2‐StACE1‐SP and pSUC2‐Avr1b‐SP constructs. Transformants were diluted and screened on CMD−W (dropout−Trp) medium (0.67% YNB, 0.075% dropout−Trp supplement, 2% sucrose, 0.1% glucose, and 2% agar) and YPRAA medium plates (1% yeast extract, 2% peptone, 2% raffinose, and 2 µg/ml antimicyn A). Invertase enzymatic activity was detected based on the reduction of TTC to the insoluble red‐coloured 1,3,5‐triphenylformazan. For liquid medium assay, the transformants were cultured in liquid CMD−W medium to OD_550_ = 0.5. Then, approximately 1.5 ml of cell suspension was mixed with colour reaction buffer (250 µl of 10 mM acetic acid–sodium acetate buffer pH 4.7, 500 µl of 10% wt/vol sucrose solution, and 750 µl of sterile distilled water) at 37℃ for 10 min. The samples were then centrifuged at 12,000 × *g* for 1 min, 100 µl of the supernatant was mixed with 900 µl of 0.1% TTC solution (i.e., 2% TTC stock solution diluted with 1 M NaOH to 0.1% TTC), and the mixtures were incubated at room temperature for 5 min. The activity of the signal peptide was determined based on the observation of changes of colour (Wang et al., 2021).

### 4.10 Agroinfection assay in *N. benthamiana*

The *StACE1* gene was amplified from *S. turcica* cDNA and cloned into the PVX vector pGR107. The recombinant plasmid was introduced into *A. tumefaciens* GV3101 by heat shock. For cell death induction experiments, *A. tumefaciens* carrying the respective recombinant plasmids was cultured in Luria Bertani (LB) medium supplemented with 25 mg/L rifampicin and 50 mg/L kanamycin at 28℃, with shaking at 200 rpm, for 48 h. To infect tobacco plants, the bacteria were washed three times with acetosyringone buffer (0.96 g/L trisodium phosphate, 9.6 g/L 2‐[*N*‐morpholino]ethanesulfonic acid, 5 g/L glucose, and 100 µl/L 1 M acetosyringone), suspended in the buffer to OD_600_ = 0.5, and used to infiltrate the leaves of 4‐week‐old *N. benthamiana* plants using a syringe without the needle attached (Situ et al., 2020). Disease symptoms were monitored visually and photographs were taken after 3–8 days. The experiment was repeated at least three times.

## CONFLICT OF INTEREST

The authors declare that they have no conflict of interest.

## Supporting information


**FIGURE S1** Bioinformatics analysis of glycosyl hydrolase (GH) family proteins. (a) Phylogenetic analysis of GH families in *Setosphaeria turcica*. GH families have been conserved during evolution in different phytopathogenic fungi, such as *Alternaria*, *Bioplaris*, and *Magnaporthe*, known causal agents of plant disease. (b, c) Domain architecture of GH proteins from different families. Conserved motifs of three GH proteins predicted using the NCBI and Pfam databases are shown. All GH proteins analysed exhibit two motifs: signal peptide (sp) and GH12/GH28/GH74 conserved domains. Three‐dimensional structures of StGH12, StGH28, and StGH74 were predicted using the Swiss model

**FIGURE S2** RT‐qPCR analysis of the expression of appressorium‐coupled genes. Genes for TIR5, p450, and FAD‐domain proteins were significantly up‐regulated during appressorium induction by cellophane and maize leaves

**TABLE S1** Primers used in the current study

**TABLE S2** *Setosphaeria turcica* genes analysed in the current study

## Data Availability

The data that support the findings of this study are available from the corresponding author upon reasonable request.
