# Supplementary figures and images for: Novel factors contributing to fungal pathogenicity at early stages of Setosphaeria turcica infection
Source: Mol Plant Pathol. 2021 Oct 10;23(1):32–44. doi: 10.1111/mpp.13140 (PMC8659557; doi:10.1111/mpp.13140)

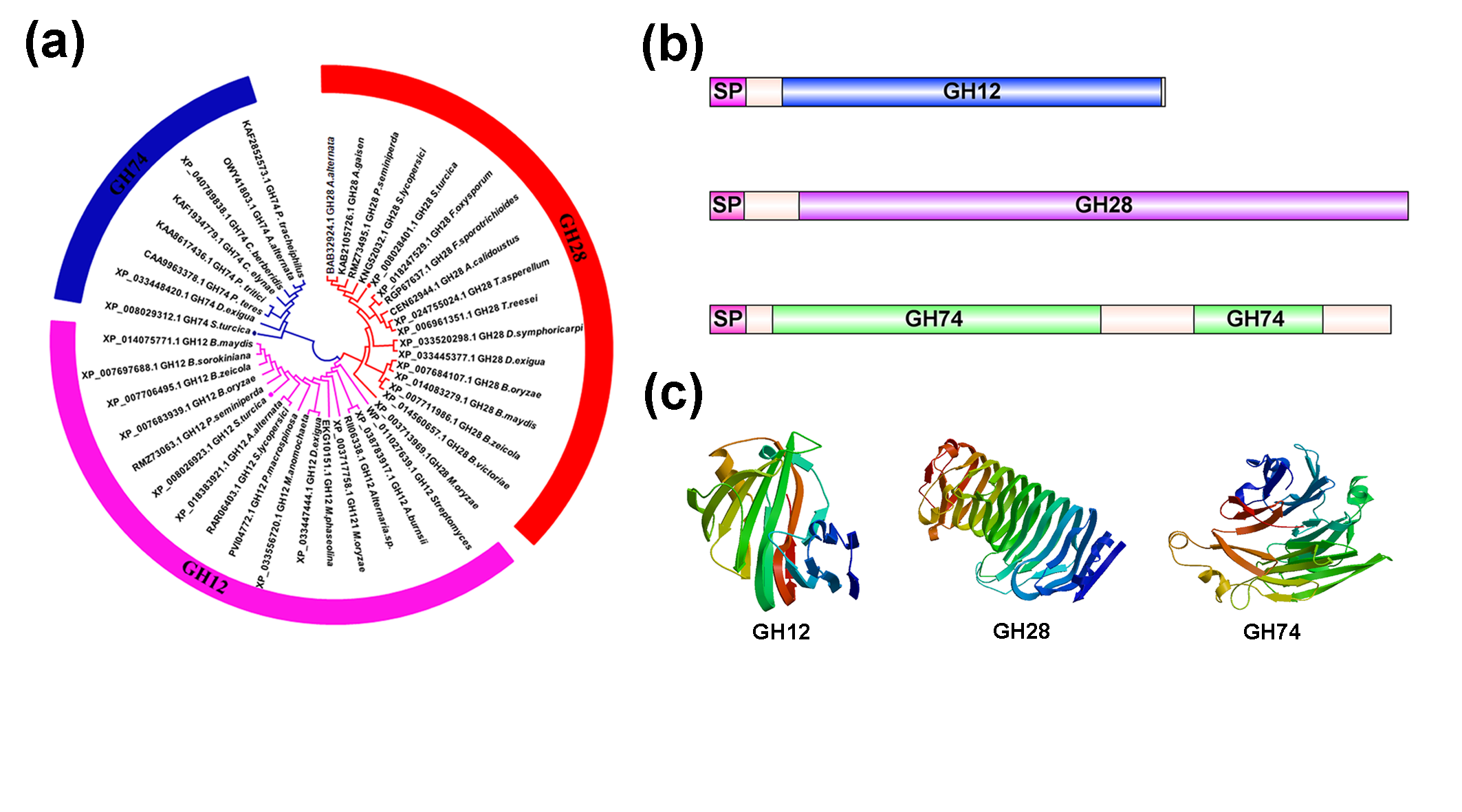

Supplement: Supplementary file 1 — FIGURE S1 Bioinformatics analysis of glycosyl hydrolase (GH) family proteins. (a) Phylogenetic analysis of GH families in Setosphaeria turcica. GH families have been conserved during evolution in different phytopathogenic fungi, such as Alternaria, Bioplaris, and Magnaporthe, known causal agents of plant disease. (b, c) Domain architecture of GH proteins from different families. Conserved motifs of three GH proteins predicted using the NCBI and Pfam databases are shown. All GH proteins analysed exhibit two motifs: signal peptide (sp) and GH12/GH28/GH74 conserved domains. Three‐dimensional structures of StGH12, StGH28, and StGH74 were predicted using the Swiss model [file MPP-23-32-s002.tif]

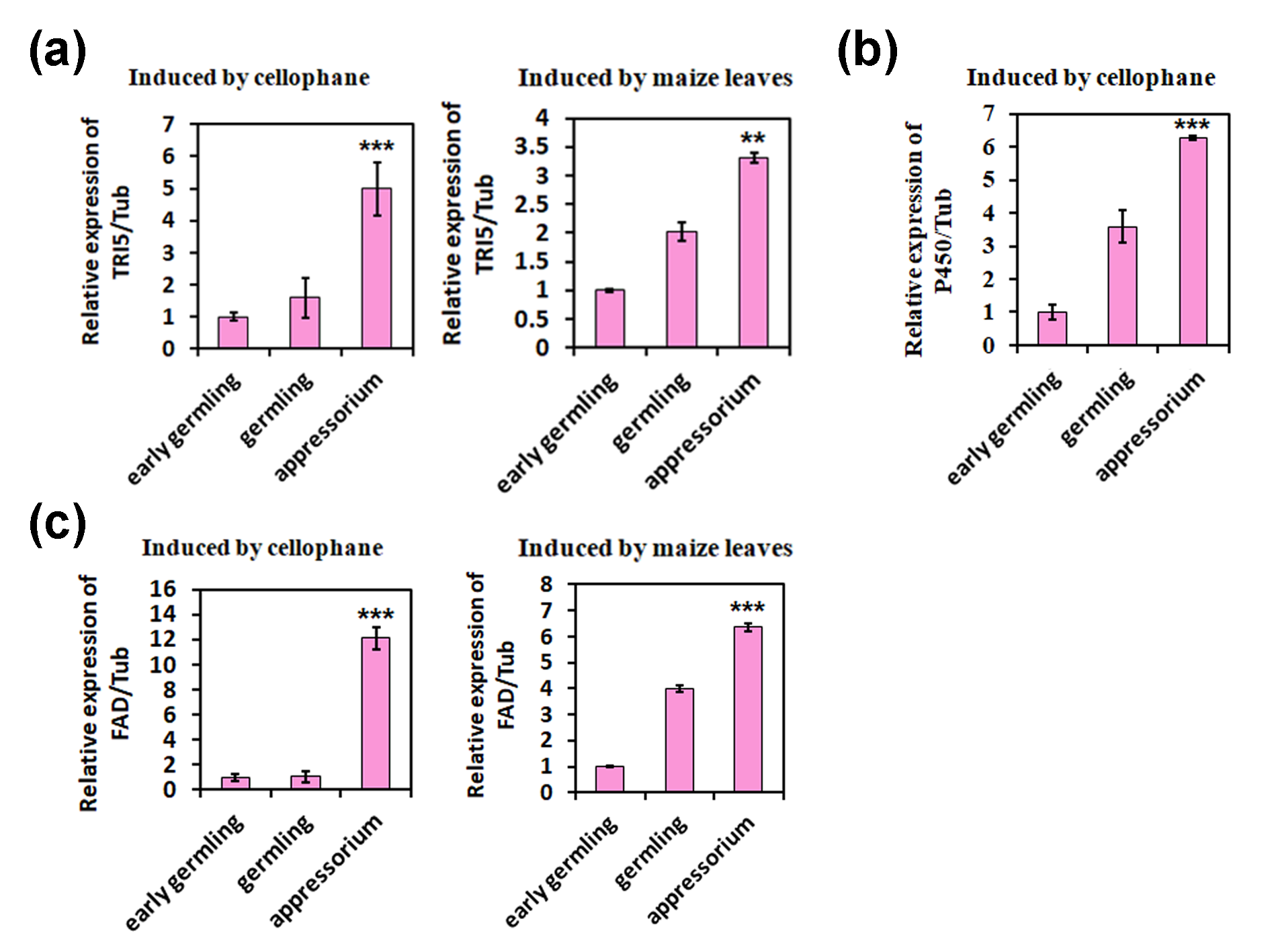

Supplement: Supplementary file 2 — FIGURE S2 RT‐qPCR analysis of the expression of appressorium‐coupled genes. Genes for TIR5, p450, and FAD‐domain proteins were significantly up‐regulated during appressorium induction by cellophane and maize leaves [file MPP-23-32-s004.tif]
