# Supplementary material for: Novel factors contributing to fungal pathogenicity at early stages of Setosphaeria turcica infection
Source: Mol Plant Pathol. 2021 Oct 10;23(1):32–44. doi: 10.1111/mpp.13140 (PMC8659557; doi:10.1111/mpp.13140)
Supplement: Supplementary file 3 — TABLE S1 Primers used in the current study [file MPP-23-32-s001.pdf]

Table S1 Primers used in this study.

Primer name	Sequence (5'-3')
SLM2-del-F1	GGTACCGGGCCCCCCCTCGAGATGCTTGGCTTGGAGATGGG
SLM2-del-R1	CAATATCATCTTCTGTCGAGGAATTCTGTGGGTGTATAGGGATGCG
SLM2-del-F2	GTACCGAGAAGGATCCTGCAACATGAAGGGATGAAAAATTGC
SLM2-del-R2	GAACAAAAGCTGGAGCTCTAGCCGTTGCCCAGCAGCATAGC
ACE1-del-F1	GTCGACGGTATCGATAAGCTTTCGCAAACTCCAGAGAAGCTC
ACE1-del-R1	GCTCACCATGATATCAAGCTTATTAGATAGGTCAGCAGTAAAC
ACE1-del-F2	ACCAGCCCCTGGGTTGGATCCATGCGTTACCGAGGGACAG
ACE1-del-R2	CGCTCTAGAACTAGTGGATCCATCCATTTTCTCTATTATCTC
SLM1-qPCR-F	TCTCCATAGGGCCTACCTCA
SLM1-qPCR-R	ATGGCAATTGGGCCGTTAC
SLM2-qPCR-F	CCAAATGGCACTTTACTAGC
SLM2-qPCR-R	ATGGTAAGCGTCTTAAGAGC
GH12-qPCR-F	AGAAGCGTGCTGACTTTTGC
GH12-qPCR-R	TTGACTTGTCCGTGGGTACC
GH28-qPCR-F	AGGTTGTCAGCATCAACGGTG
GH28-qPCR-R	CTCTGGAACTCACATCTCCT
GH74-qPCR-F	ATGAAGCTGCTTACCGCCTTG
GH74-qPCR-R	TCGGTAGTGCGGATGATGGTG
ACE1-qPCR-F	CAGCAACAGATATATCACG
ACE1-qPCR-R	CGAAGTACAGTACAAGTCTCG

TRI5-qPCR-F	ATGGGTCATTCACGCCACGA
TRI5-qPCR-R	CCTAACTGGCCTACCCATCT
P450-qPCR-F	TATGCTCCTGCCGTCATGAT
P450-qPCR-R	CTGATTCAGATGAGAGCGAG
FAD-qPCR-F	AAGTGACATACCAACTTGCAG
FAD-qPCR-R	TTTGACCATGTAGTTGTCTGd
Tublin-qPCR-F	GGGAACTCCTCACGGATCTTG
Tublin-qPCR-R	TAACAACTGGGCAAAGGGTCA
GH12-GST-F	TCTGTTCCAGGGGCCCCTGGGATCCATGAAGCTCTCTACATTCATTGCTG
GH12-GST-R	AGTCGACCCGGGAATTCGGGGATCCCTATTTCTCAGACAGGCTGTACTCG
GH28-GST-F	TCTGTTCCAGGGGCCCCTGGGATCCATGGTTGTCCTAACTGCTGGCATCTTC
GH28-GST-R	AGTCGACCCGGGAATTCGGGGATCCTTACAAGCACGCGGCAGCGC
GH74-GST-F	TCTGTTCCAGGGGCCCCTGGGATCCATGAAGCTGCTTACCGCCTTG
GH74-GST-R	AGTCGACCCGGGAATTCGGGGATCCTTAGCGAAAGCTAAGCACGC
pSUC2-ACE1-F	CGGAATTTTAATTAAGAATTCATGCGTTACCGAGGGACAG
pSUC2-ACE1-R	ACTATAGGGAGAACCTCGAGAATACAATACCAGGCAGTCAC
pSUC2-chk-F	CCTCGTCATTGTTCTCGTTCCCTT
pSUC2-chk-R	GGTGTGAAGTGGACCAAAGGTCTA
pGR107-ACE1-F	CTAGAACTAGTGGATCCCCCGGGTGGTATTGTATTACCAAAGAAGGGACTTT
pGR107-ACE1-R	C TCGCCCTTGCTCACCATCCCGGGCTAAACACACGTAGCCATGTCG
pGR107-chk-F	CAATCACAGTGTTGGCTTGC
pGR107-chk-R	GACCCTATGGGCTGTGTTG
