# Supplementary material for: Novel factors contributing to fungal pathogenicity at early stages of Setosphaeria turcica infection
Source: Mol Plant Pathol. 2021 Oct 10;23(1):32–44. doi: 10.1111/mpp.13140 (PMC8659557; doi:10.1111/mpp.13140)
Supplement: Supplementary file 4 — TABLE S2 Setosphaeria turcica genes analysed in the current study [file MPP-23-32-s003.pdf]

Table S2 Information of *S. turcica* genes in this study.

Name	GenBank Accession	JGI (protein ID)	KOG	*S. cerevisiae* homologous
SLM1	XP_008022589.1	146662	Phosphatidylinositol 4,5-bisphosphate-binding protein, SLM1	SLM1
SLM2	XP_008024338.1	168684	Phosphatidylinositol 4,5-bisphosphate-binding protein, SLM2	SLM2
GH12	XP_008026923.1	20746	Glycosyl hydrolase family 12	-
GH28	XP_008028401.1	92363	Glycosyl hydrolases family 28	-
GH74	XP_008029312.1	164939	Oligoxyloglucan reducing end-specific cellobiohydrolase	-
ACE1	-	1195465	Unknown	-
MSH2	XP_008027367.1	90274	Chromosome segregation ATPase DNA polymerase III subunits; DNA mismatch repair protein 2	MSH2
TRI5	XP_008023317.1	38037	Trichodiene synthase (TRI5)	-
P450	XP_008023399.1	159901	Cytochrome P450	-

FAD	XP_008026371.1	110485	FAD binding domain, 6-hydroxynicotinate 3-monooxygenase	-
TUB2	XM_008032318.1	165677	Tubulin	TUB2
-	XP_008022480.1	37488	Cytochrome c oxidase subunit vib protein	-
KSP1	XP_008023983.1	107556	Serine/threonine-protein kinase, Ksp1	
-	XP_008029820.1	165213	Homoserine dehydrogenase	-
DUF1996	XP_008020432.1	85861	DUF1996	-
-	XP_008027676.1	137719	PPR repeat	-
-	XP_008030944.1	181691	Unknown	-
